# Exploration and Research of Human Identification Scheme Based on Inertial Data

**DOI:** 10.3390/s20123444

**Published:** 2020-06-18

**Authors:** Zhenyi Gao, Jiayang Sun, Haotian Yang, Jiarui Tan, Bin Zhou, Qi Wei, Rong Zhang

**Affiliations:** Department of Precision Instrument, Engineering Research Center for Navigation Technology, Tsinghua University, Beijing 100084, China; gaozy17@mails.tsinghua.edu.cn (Z.G.); sun-jy17@mails.tsinghua.edu.cn (J.S.); yang-ht17@mails.tsinghua.edu.cn (H.Y.); tjr16@mails.tsinghua.edu.cn (J.T.); weiqi@tsinghua.edu.cn (Q.W.); rongzh@mail.tsinghua.edu.cn (R.Z.)

**Keywords:** human identification, inertial data, classification experiments, feature visualization

## Abstract

The identification work based on inertial data is not limited by space, and has high flexibility and concealment. Previous research has shown that inertial data contains information related to behavior categories. This article discusses whether inertial data contains information related to human identity. The classification experiment, based on the neural network feature fitting function, achieves 98.17% accuracy on the test set, confirming that the inertial data can be used for human identification. The accuracy of the classification method without feature extraction on the test set is only 63.84%, which further indicates the need for extracting features related to human identity from the changes in inertial data. In addition, the research on classification accuracy based on statistical features discusses the effect of different feature extraction functions on the results. The article also discusses the dimensionality reduction processing and visualization results of the collected data and the extracted features, which helps to intuitively assess the existence of features and the quality of different feature extraction effects.

## 1. Introduction

Inertial data are data obtained by inertial sensors (gyros, accelerometers, magnetometers), including triaxial acceleration, triaxial angle or angular velocity and attitude angles, etc. With the development of MEMS (Micro-Electro-Mechanical System) technology, inertial data can be measured by a magnetometer and an IMU (Inertial Measurement Unit), which is a combination of gyroscopes and accelerometers. Magnetometers and IMUs have been widely used in wearable sensors due to their small size, light weight, low power consumption, and portability [1,2]. Due to the uniqueness of the creature’s posture during the movement, the inertial data can be used to distinguish the current movement state of the creature [3,4], which has become more and more widely used in medical rehabilitation, virtual reality, somatosensory games and other fields [5,6]. The unique movement characteristics of biological individuals can also be used as biometrics to identify biological identities. In the field of intelligent surveillance, compared to face recognition, fingerprint recognition, and iris recognition, which are limited by resolution, space, and distance, this type of identity recognition based on inertial data has higher concealment and is difficult to prevent [7,8]. At the same time, IOS or Android-based smart phones, smart bracelets, smart watches, etc., have integrated the magnetometer and IMU. With the improvement of computing performance, they are capable of acquiring and processing individual motion data, thereby analyzing and identifying the current motor behavior and physiological status of users.

The research methods for obtaining behavior characteristics and analysis based on inertial data are relatively mature, and there are many related studies. An activity recognition system was proposed in [9], which can determine which daily activity the current behavior belongs to, such as running, walking, standing, and sitting down, lying, falling, etc. In this study, researchers used a mobile phone to obtain triaxial acceleration data, and applied a Multi-Layer Perceptron (MLP) algorithm to classify the data. A similar study is on gait authentication and identification using a wearable accelerometer sensor [10], in which researchers performed statistical analysis on inertial data and determined the type of current behavior. This is a typical application for inertial data used in gait analysis. Another example was proposed in [11]. The researchers used IMU-based inertial data for joint axis and position identification, as well as flexion and extension joint angle measurement.

In addition to gait recognition based on inertial data, feature extraction and processing of inertial data can also be performed for specific application scenarios or other kinematic features. Paper [12] reported a case of life scene analysis based on a triaxial accelerometer. Using the acceleration data, researchers can identify that the current status is driving, exercising or other states. This is the processing and classification of inertial data, while expanding the scope of research goals. Inertial data can also play a role in observing the details of motion. In [13], researchers can use the angular velocity and acceleration data during joint movements to obtain the state of movement of various parts of the body such as the neck, shoulders, and waist. This analysis method has been applied in scenarios such as gesture recognition [14] and baseball swing posture assessment [15].

Inertial data can be used to describe motion characteristics, and, as biological characteristics, motion characteristics have also been applied in human identification. James et al., [16] first proposed that people’s identity can be judged by walking gestures. Afterwards, researchers performed gait characterization and feature extraction through image data, and performed identity classification. Su et al., proposed a novel method [17] to attain limb angle information by analyzing the variation of silhouette width without needing the human body model. Foster et al., proposed a method [18] for gait analysis and gender identification using area-based metrics. Lu et al., [19] proposed a gait recognition method for human identification based on ICA and a fuzzy support vector machine (SVM) through the fusion of multiple views. The above studies are based on image information for gait estimation and identity recognition. There are many studies on gait estimation using inertial data, but there are no research results in terms of human identity recognition with inertial data.

Before introducing the exploration process, the main methods of human identification are summarized in Table 1. It can be seen from the table that the main method is to extract identity information from the image. Image-based processing schemes have a lot of related research work, but they will be limited by the environment and data collection process. The solution discussed in this paper has no relevant concerns. In contrast, there is no relevant reliable research and it is necessary to discuss the feasibility of this method and explore it from scratch.

This paper mainly discusses two issues, one is whether human identity can be recognized by analyzing inertial data; the second is how to extract human identity-related features from inertial data. These two issues are related. The first one focuses on the distinguishability of inertial data itself for human identification and the distinguishability of the data after processing, to clarify the possibility of human identification based on inertial data. The second problem is an extension of the first problem, which aims to find a way of using feature extraction to explore the feasibility of identity recognition based on inertial data in large-scale samples. When the number of people to be identified is large, feature-based matching is more appropriate than classification methods. Exploring the feature extraction method and feature distinguishability is the main concern of the second issue.

The article is organized as follows. Section 2 discusses the problem to be solved and the research method. Section 3 provides the verification results of inertial data distinguishability. Section 4 presents the classification experiment results based on different feature extraction methods. Finally, Section 5 provides conclusions and future research plans.

## 2. Problem Statement and Data Preprocessing

### 2.1. Description of Research Content and Dataset

In order to study the possibility of human identity recognition based on inertial data, the first study is about whether the data itself is distinguishable from human identities. Data dimensionality reduction analysis was performed on the data to observe the data distinguishability from the perspective of data visualization, and a classification algorithm was applied to further verify its distinguishability. The experimental results show that the data itself is indistinguishable, so the second part is to try to find methods to extract and classify the features related to human identities. This part verifies whether the identity recognition is feasible for the selected inertial data set and which feature extraction scheme should be selected.

**Figure 1:**
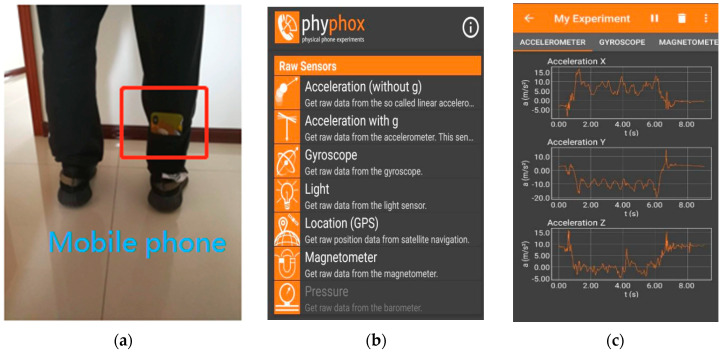
Data collection method. (**a**) A mobile phone tied to the leg while data collecting; (**b**) operation interface for sensor selection; (**c**) data acquisition interface for accelerometer.

Before the data preprocessing content and the experimental details are introduced, the collected inertial data and the data acquisition scheme are described. In the experiment, 10 subjects were selected to collect walking data using the inertial sensors built into their mobile phones. The mobile phone was tied to the leg as shown in Figure 1a. The collected data includes the X, Y, Z three-dimensional data output by gyroscopes, accelerometers and magnetometers. The gyroscopes output angular velocity information, the accelerometers output acceleration information, and the magnetometers output attitude angle information. The phyphox software [20] was used for data collection with a sampling frequency of 100 Hz and a sampling time of about 30 s. The software is a toolkit for physical phone experiments provided by RWTH Aachen University, and can be downloaded from the application gallery in the phone. Figure 1b,c show the software operation interface and data acquisition interface, respectively. In the experiment, the collected data set contains the inertial data of 10 people in a total of 34,440 groups, with 9 data in each group.

### 2.2. Data Preprocessing

The inertial data collected by the mobile phone sensor not only contains the feature information of each pedestrian, but also various noise interferences [21]. In order to accurately extract the required features in the subsequent recognition process, the data preprocessing is conducted [22]. The preprocessing steps include noise elimination and the process of normalization.

In the experiment, moving average filtering [21] was applied, which is easy to implement and has high robustness. The arithmetic average of the data at each sampling time, the sampling data at the previous two times, and the data at the next two sampling times, is taken as the processed sampling data at that time. The process can be expressed by the following formula:

(1) X(k)=(D(k+2)+D(k+1)+D(k)+D(k−1)+D(k−2))/5, (k=1,2, …,N)

where k represents one sample time, N represents the number of sampling points and X(k) represents the filtered data.

Different kinds of inertial data have different data ranges, and the change of the data can better reflect biological characteristics than the data range. The normalization method [23] is used to set all data to the range of [0, 1] to normalize the feature scale and make possible features distinctive. The method can be expressed by the following formula:

(2) $$X_{s}=\frac{X-X_{min}}{X_{max}-X_{min}}$$

where X is the data at a certain time, $X_{min}$ is the minimum value of the data in the continuous time range, $X_{max}$ is the maximum value, and $X_{s}$ is the data after normalization.

All data will be processed for noise elimination and normalization before further processing. Subsequent research and experiments will also be performed on the basis of data preprocessing.

## 3. Verification of Inertial Data Separability

The purpose of data separability research is to confirm that biometric information related to identity may be extracted from the time series of data, but not from data at a certain sampling time. A dimensionality reduction method named Principal Component Analysis (PCA), and visual analysis, were performed on the data directly, and a classification algorithm named the K-Nearest Neighbor (KNN) algorithm was applied, without considering the time series characteristics, to further verify that the features are contained in the time series if the features exist.

### 3.1. PCA-Based Data Separability Verification

The PCA algorithm is an effective method to reduce the dimension of feature space, which was first proposed by K. Pearson in 1901 [24]. It is an important statistical method to extract fewer features from multiple features, on the premise of accurately expressing the object features [25]. It can project high-dimensional data into low-dimensional space and form a group of new principal components, which are independent of each other and have no redundancy, so as to achieve the purpose of dimension reduction. It is often used for feature extraction and data visualization.

The inertial data collected in the experiment contains three-axis acceleration, angle, and attitude angle information, that is, each set of data is a 9-dimensional vector. The data was reduced to two-dimensional data after applying the PCA algorithm, and the data was visualized. If the inertial data are separable, different types of data should be located at different positions on the plane, and different types of data can be distinguished according to the position.

The dimensionality reduction process can be simplified into the following expression:

(3) $$Y_{N\times 2}=X_{N\times 9}\cdot A_{9\times 2}$$

where N represents the number of groups of data, each group of raw data contains nine elements. A is the transformation matrix that needs to be calculated in the PCA algorithm, and X and Y represent the data before and after the transformation, respectively. The calculation of the transformation matrix A can refer to the method in [26].

**Figure 2:**
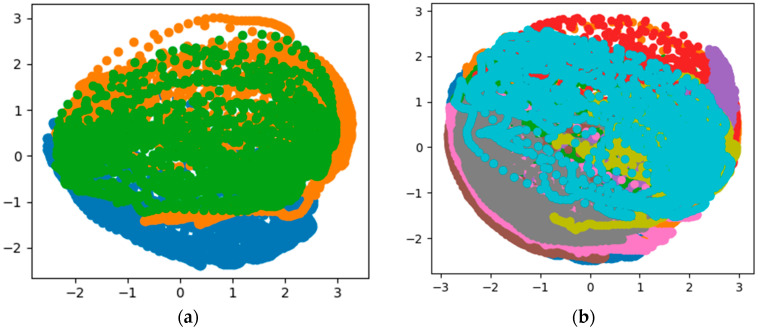
Data visualization of Principal Component Analysis (PCA) results. (**a**) Results of 3 people; (**b**) results of 10 people.

Figure 2 shows the visualization results of the PCA dimensionality reduction on the inertial data of three people, and the visualization results of the inertial data of 10 people.

The coordinate axis has no actual physical meaning, and different colors belong to different people’s data. From the visualization results of PCA dimensionality reduction, the inertial data of different people are mixed after dimensionality reduction, which is difficult to distinguish, so the data itself is not separable according to the data visualization results.

### 3.2. KNN-Based Data Separability Verification

In order to further verify the indistinguishable characteristics of the data and explain the necessity of feature extraction, the KNN classification algorithm was used for data separability analysis.

The principle of KNN is to find the K training samples closest to the testing sample based on a certain distance measurement, and then the category of samples will be predicted based on the information of K “neighbors”. Generally, the category with the most frequent occurrence in K samples is recognized as the prediction result [27].

In the experiment, Euclidean distance is used to describe the distance between two samples. Each set of sample data is a vector with nine elements. The Euclidean distance of samples $Y_{i}$ and $Y_{j}$ is defined as follows:

(4) $$D_{i,j}=\sqrt{\overset{9}{\underset{k=1}{\sum }}{\left(Y_{i,k}-Y_{j,k}\right)}^{2}}$$

where $Y_{i,k}$ represents the k-th element of sample $Y_{i}$.

**Figure 3:**
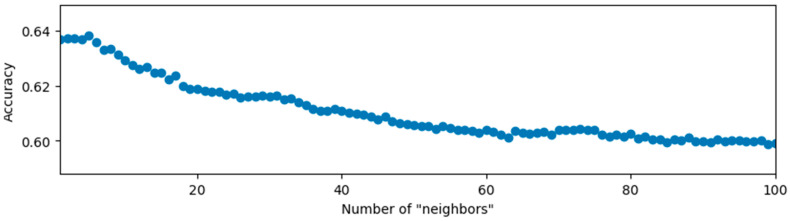
Relationship between classification accuracy and the number of “neighbors”.

The first 60% of the entire data set is used as the training set and the last 40% is used as the test set. The method of data set division has also been used in subsequent studies. The number of “neighbors” of the KNN algorithm is set from 1 to 100. The classification accuracy is defined as the number of correctly classified test samples divided by the total number of test samples, and the results are shown in Figure 3.

From the experimental results, when the number of “neighbors” is five, the classification accuracy is the highest, and the result is 0.6384.

According to the data reduction and visualization results of PCA, the inertial data, without considering the time series changes, is not distinguishable and cannot be used to judge the identity of others. After applying the KNN classification algorithm to the inertial data, the highest accuracy rate obtained is only 63.84%, further verifying the indistinguishability of the data itself. This means that identity-related features should be extracted from the changes of the data if they exist.

## 4. Classification Experiments Based on Feature Extraction

If the changes between the inertial data at different times are not considered and the identification accuracy is based on the data at the sampling time, the obtained classification accuracy is low. This part serves as a control experiment and will show the classification results after extracting information from a period of inertial data.

### 4.1. Feature Extraction Based on Statistical Data and Identity Identification Based on SVM Algorithm

This section will introduce the experimental results of extracting statistical features and using the SVM algorithm for classification. Since the data collected by the sensor is inertial data in continuous time, the sliding window technique [28] is used to segment the data and extract statistical features from the data in the window. The number of overlapping sampling points when the window slides is set to 1, and the effect of the size of the window on the classification results was studied in the experiment. Each window of data extracts 18 statistical features. The main concern in the experiment is not whether the most suitable statistical features are selected, but to verify the separability of the feature based on the selected statistical features.

The statistics in the time domain and frequency domain of the data in each sliding window are calculated as statistical features. In the time domain, the average, variance, standard deviation, maximum, minimum, zero-crossing times, the difference and mode between the maximum and minimum values [29,30] are calculated and used as time-domain statistics features. In the frequency domain, the Fast Fourier Transform (FFT) algorithm was applied to obtain frequency domain information, and to extract DC components, average amplitude, amplitude variance, amplitude standard deviation, amplitude deviation, amplitude kurtosis, shape average, shape variance value, shape standard deviation and shape kurtosis [31,32].

**Figure 4:**
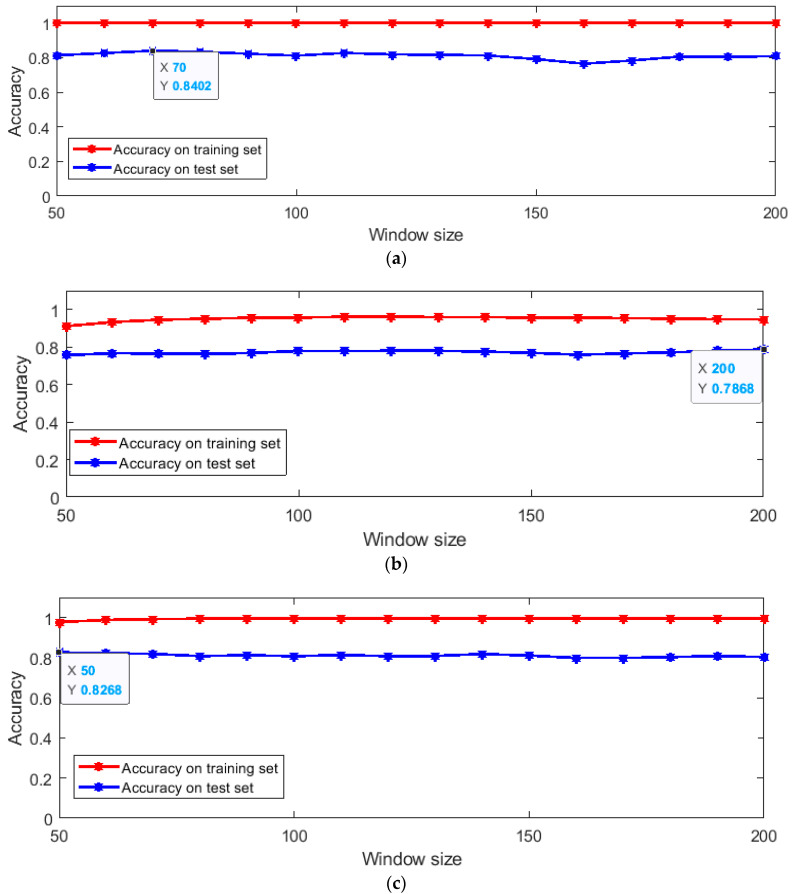
Support vector machine (SVM) classification results of different kernel functions. (**a**) Results of linear kernel function; (**b**) results of polynomial kernel function; (**c**) results of radial basis kernel function.

For the extracted statistical features, the SVM algorithm is used for feature classification. SVM is a supervised classifier, and the basic principle is to obtain the separation hyper plane with the largest geometric interval of different types of data [33]. The SVM algorithm is widely used in pattern recognition problems such as portrait recognition and text classification [34]. Kernel function is always used in the SVM algorithm to map linearly inseparable data to another spatial domain, making it linearly separable. Commonly used kernel functions include linear kernel functions, polynomial kernel functions and radial basis kernel functions [35,36]. In the experiment, the above three kernel functions were used to conduct SVM classification experiments, respectively. The experimental results are shown in Figure 4 and the size of the window is set from half to twice the sampling frequency.

The use of different kernel functions is to avoid the effect of whether the statistical features are linearly separable on the results, and pay more attention to the distinguishable features of the statistical features. The maximum accuracy of different kernel functions on the test set is also marked in Figure 4. According to the experimental results, the classification results using linear kernel functions are the best on the test dataset. When the window size is 70, the accuracy rate on the training set is 1, and the accuracy rate on the test set is 0.8402. The result on the test set is better than the result of the KNN algorithm, which shows that the statistical characteristics that contain information about changes in inertial data are, to some extent, more reflective of information related to human identity.

**Figure 5:**
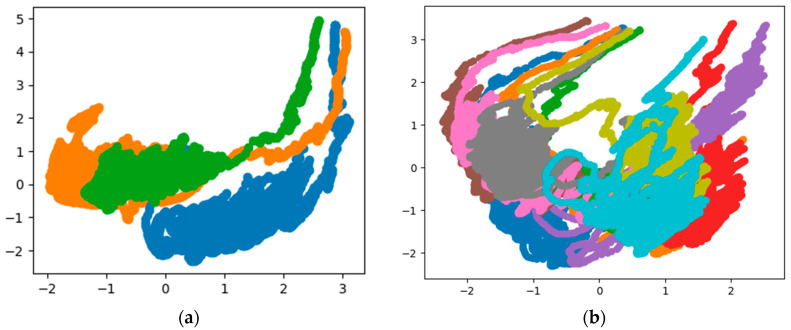
Data visualization of PCA results for statistical features. (**a**) Results of 3 people; (**b**) results of 10 people.

Dimensionality reduction and visualization analysis of statistical features were performed by applying the PCA algorithm. The purpose is to intuitively observe whether the separable characteristics of statistical features have improved, to some extent, compared to the inertial data. The results are shown in Figure 5.

Compared with Figure 2, the visualization results show that the distinguishability between different colors or different people has improved. From the visualization results and the results of the SVM algorithm, statistical characteristics can be used as the basis for human identification on the collected data set, which can achieve 84.02% accuracy. If the parameters in the algorithm are adjusted, this result may be better. To further verify the features related to human identity information present in the inertial data, the next section will introduce another black box-like feature extraction method to obtain even higher accuracy on the test data set, as well as better data dimensionality reduction and visualization results.

### 4.2. Machine Learning-Based Identity Recognition

This section will introduce the experimental results of training a neural network as a feature extraction function and the classification results based on the extracted features. The neural network used in the study is MLP, and the network parameter values are obtained through training, so that the network serves as the fitting function of the specific input and output system [37].

**Figure 6:**
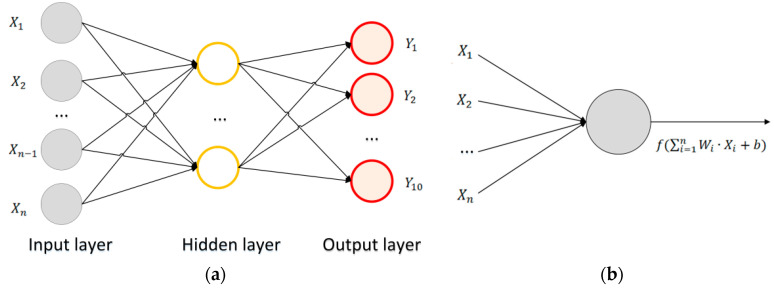
Model of Multi-Layer Perceptron (MLP) and neurons. (**a**) MLP model composed of neurons; (**b**) mathematical model of a neuron.

The model of MLP is shown in Figure 6a, which is composed of neurons, and the neurons form the input layer, hidden layer and output layer. The neuron model is shown in Figure 6b. Assuming that the input layer is represented by a vector X, the output of a neuron in the hidden layer is f(W·X+b), in which W is the weight (also called the connection coefficient), b is the output bias and f is the activation function [37].

In the experiment, the Rectified Linear Unit (ReLU) [38] was used as the hidden layer activation function, and the softmax function [38] was used as the output layer activation function. The hidden layer acts as a feature extraction function, and the output layer acts as a classifier to participate in network optimization and classification accuracy calculation. All the parameters of MLP are the connection weights and output bias between the various layers. The process of solving these parameters is the training process of the network. The training process is an iterative learning process, which uses the Adam [39] optimization algorithm.

**Figure 7:**
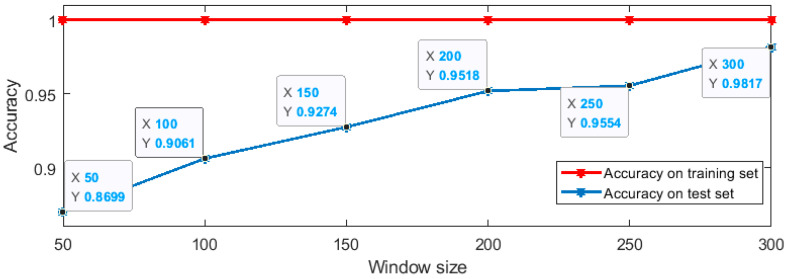
MLP classification results of different window size.

In the experiment, the sample data are obtained by sliding window, and the data in the sliding windows was directly fed into the network for parameter fitting, which is different from the classification experiment of extracting statistical features from the data in the sliding window. In the experiment, different sizes of sliding window were selected and the results are shown in Figure 7.

According to the experimental results, the accuracy of the larger sliding window on the test set is higher. When the size of the window is 300, the classification accuracy on the test set is 0.9817. In order to compare with the classification results of statistical features, SVM classification based on statistical features was also performed when the window size was 300. When the kernel function is the radial basis function, the classification scheme based on statistical features gets the highest accuracy on the test set, with a value of 0.8199, which is less than the accuracy of the MLP scheme.

**Figure 8:**
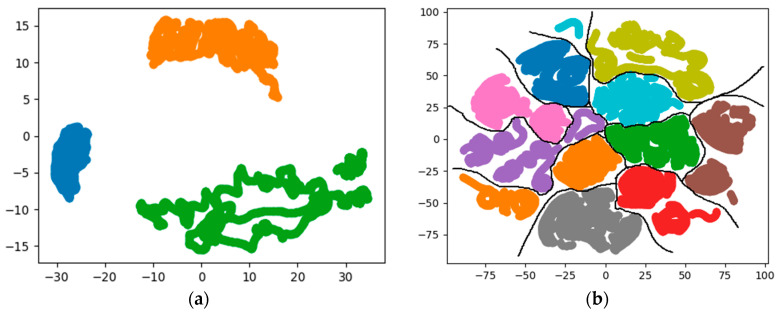
Data visualization after dimensionality reduction for the extracted features by MLP. (**a**) Results of 3 people; (**b**) results of 10 people.

The output of the MLP hidden layer is regarded as the extracted features related to human identity, and dimensionality reduction and visualization analysis of the extracted features were also performed. The results are shown in Figure 8.

Compared with Figure 2 and Figure 5, the visualization results show that that after feature extraction, the characteristics between different people can be clearly distinguished. From the visualization results and the accuracy of the algorithm, the feature function fitted using MLP can be used as the basis for human identification on the data set used. The extracted features are classified using the SVM algorithm with a radial basis kernel function. The accuracy on the test set is 0.9881. Excluding the influence of the classifier, the results indicate that the features extracted by MLP can reflect information related to human identity more accurately than statistical features.

## 5. Conclusions

Through analysis and experiments, this paper confirms that the collected data can be used for human identification based on inertial data. According to whether or not to extract features, different experiments were carried out. The results show that the information related to human identity is included in the changes of inertial data, and this information needs to be extracted. Statistical functions and a neural network fitting function (MLP) were used for feature extraction; the classification results based on the latter reached 98.17%, further indicating that the features related to human identity inertial data in the experiment can be extracted through a feature extraction function, and the visual results of the extracted features indicate that the features can be used to distinguish people’s identities.

Compared with the image processing-based identity recognition studies, the proposed scheme is not affected by the environment, while image processing schemes generally need to remove the background and are affected by the environment. The highest accuracy rate of image-based recognition schemes under laboratory conditions is 95.0% [19], which is less accurate than the proposed method. Freedom from background and environmental interference is an advantage of the proposed scheme, but the lack of a clear feature modeling method is a disadvantage.

The paper did not find a clear expression of the most suitable feature extraction function, but verified the feasibility of the neural network on the data set to fit the feature extraction function. From the experimental results, the inertial data contains features related to human identity. In order to further verify the role of features in identifying human identity, it is considered to expand the size of the data set to distinguish more people. When recognizing more human identities, simplifying the recognition as a classification problem will no longer be applicable. Human identity matching and recognition based on feature extraction functions will be the next research content.

## Figures and Tables

**Table 1:** Summary of typical methods of human identification based on motion characteristics.

Method Category	Data Sources	Feature Extraction	Advantages	Disadvantages
Joint position changes [16]	Position of joints in the image	Statistics of positions	Simple data processing	Complex image acquisition method and low accuracy
Extract limb angle information from images [17]	Image sequence	Analyze the change in silhouette width	No human body required, high accuracy	Still background is required
Recognition using area-based metrics [18]	Image sequence	Body contour extraction and combination of masks	Simple calculation, high accuracy	Need a fixed camera position for image acquisition
Method based on machine learning	Image sequence	Body contour extraction and classification based on SVM	Feature fusion, high accuracy	Need a fixed camera position
Solutions explored and discussed in this article	Inertial data	Statistical features and network fitting features	Simple data collection, not affected by the environment, high accuracy	The method of feature extraction needs further exploration to meet the use of large-scale data
